# Effects of environmental and biotic factors on carbon isotopic fractionation during decomposition of soil organic matter

**DOI:** 10.1038/srep11043

**Published:** 2015-06-09

**Authors:** Guoan Wang, Yufu Jia, Wei Li

**Affiliations:** 1 Department of Environmental Sciences and Engineering, College of Resources and Environmental Sciences, China Agricultural University, Beijing 100193, China; 2 Key Laboratory of Mountain Surface Processes and Ecological Regulation, Institute of Mountain Hazards and Environment, Chinese Academy of Sciences, Chengdu 610041, China

## Abstract

Decomposition of soil organic matter (SOM) plays an important role in the global carbon cycle because the CO_2_ emitted from soil respiration is an important source of atmospheric CO_2_. Carbon isotopic fractionation occurs during SOM decomposition, which leads to ^12^C to enrich in the released CO_2_ while ^13^C to enrich in the residual SOM. Understanding the isotope fractionation has been demonstrated to be helpful for studying the global carbon cycle. Soil and litter samples were collected from soil profiles at 27 different sites located along a vertical transect from 1200 to 4500 m above sea level (a.s.l.) in the south-eastern side of the Tibetan Plateau. Their carbon isotope ratios, C and N concentrations were measured. In addition, fiber and lignin in litter samples were also analyzed. Carbon isotope fractionation factor (α) during SOM decomposition was estimated indirectly as the slope of the relationship between carbon isotope ratios of SOM and soil C concentrations. This study shows that litter quality and soil water play a significant role in isotope fractionation during SOM decomposition, and the carbon isotope fractionation factor, α, increases with litter quality and soil water content. However, we found that temperature had no significant impact on the α variance.

Soil organic carbon is the largest pool of terrestrial ecosystem and greatly affects global carbon cycling. The CO_2_ derived from soil organic matter (SOM) decomposition is an important source of atmospheric CO_2_. The CO_2_ released from soil respiration enriches ^12^C while the residual SOM enriches ^13^C, relative to the substrate, because of carbon isotopic fractionation during SOM decomposition^1^,^2^,^3^,^4^,^5^,^6^,^7^. Consequently, the isotopic fractionation affects the carbon isotope composition of atmospheric CO_2_ because the released CO_2_ finally goes into the atmosphere. Scientists who study global change incorporate carbon isotope data for tropospheric CO_2_, derived from an international network of stations, into atmospheric circulation models. This step is used to calculate global carbon balance and to analyze atmospheric carbon source/sink positions and quantities^8^,^9^,^10^,^11^. Thus, understanding carbon isotope fractionation during SOM decomposition can help scientists use carbon isotope data for atmospheric CO_2_ in their studies of global carbon cycling. In addition, it has been demonstrated that adding soil carbon isotope variations to carbon-dynamic models provides tighter constraints on certain model parameters having biological and environmental significance^12^,^13^. An understanding of carbon isotope fractionation during SOM decomposition can also enhance reconstructions of past environments. This can benefit studies of C_4_-plant origins and expansions in geological time that use carbon isotope records of ancient terrestrial sediments^6^,^14^,^15^,^16^,^17^,^18^.

It is well known that environmental and biotic factors affect the decomposition rate of organic matter^13^,^19^,^20^,^21^,^22^. Substrate quality q is the dominant biotic influential factor^12^,^13^,^23^,^24^. Substrate quality quantifies how easily organic carbon is used by soil microbes^12^,^13^. It can be related to plant type and is often defined using a C/N ratio, lignin content, cellulose content, and/or lignin content/N ratio^25^,^26^. The influences of environmental factors and substrate quality on decomposition have been assessed intensively^27^,^28^,^29^,^30^,^31^,^32^,^33^,^34^. However, few studies have focused on whether these factors influence carbon isotope fractionation during SOM decomposition^3^,^12^,^13^,^25^,^35^. Therefore, current study was undertaken to evaluate the effects of environmental and biotic factors on carbon isotope fractionation during SOM decomposition. In this regard we measured the carbon isotope ratios of soil and litter samples collected from soil profiles along an altitudinal gradient in the south-eastern side of the Tibetan Plateau. Furthermore, their carbon isotope fractionation (α) during SOM decomposition was estimated using an indirect method established by Poage and Feng (2004)^13^.

## Results

### Variations in C/N, lignin, and cellulose of litters with altitude

**Figure 1:**
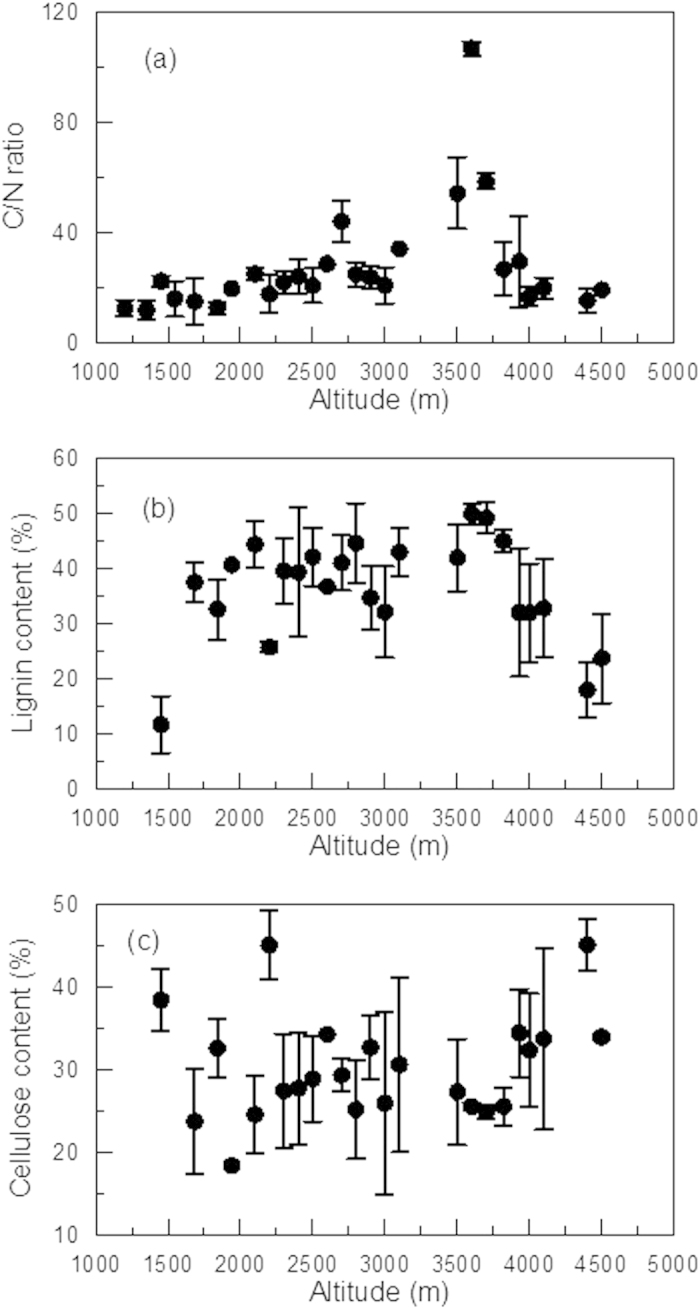
shows the variations in C/N ratios (**a**), lignin contents (**b**) and cellulose contents (**c**) of litter with altitude.

The C/N ratio of litters varied greatly and ranged from 8 to 109 (Fig. 1a). Since litters collected in this study were mainly composed of leaves *in situ*, the C/N ratio of litter depended on the vegetation types and plant species. The C/N ratio increased up to an elevation of 3600 m and then decreased at higher elevations (Fig. 1a). This observation indicates that the coniferous trees (mainly located at 2800–3600 m) had the highest C/N ratios, and the broad-leaved trees (mainly located at 1600–2800 m) and shrubs (mainly located at 1600–2800 m and 3600–4200 m) had the second highest C/N ratios. In addition, the herbaceous plants, including grasses, which were mainly composed of C_4_ species (located below 1600 m) and alpine frigid meadow vegetation (located at 4200–4600 m) had the lowest C/N ratios.

The woody plants including trees and the shrubs had much higher lignin contents than the herbaceous plants (Fig. 1b). This altitudinal pattern of lignin arose because herbaceous plants are mainly distributed at elevations less than 1600 m and in the range of 4200–4600 m. However, the latter had higher cellulose contents than the former (Fig. 1c). Correlation analysis shows that the C/N ratio of litter is significantly and positively related to the lignin content (p = 0.000) and negatively related to cellulose content of litter (p = 0.008). In addition, a significant negative correlation exists between lignin and cellulose (p = 0.000).

### Influences of environmental and biotic factors on fractionation factor α

**Figure 2:**
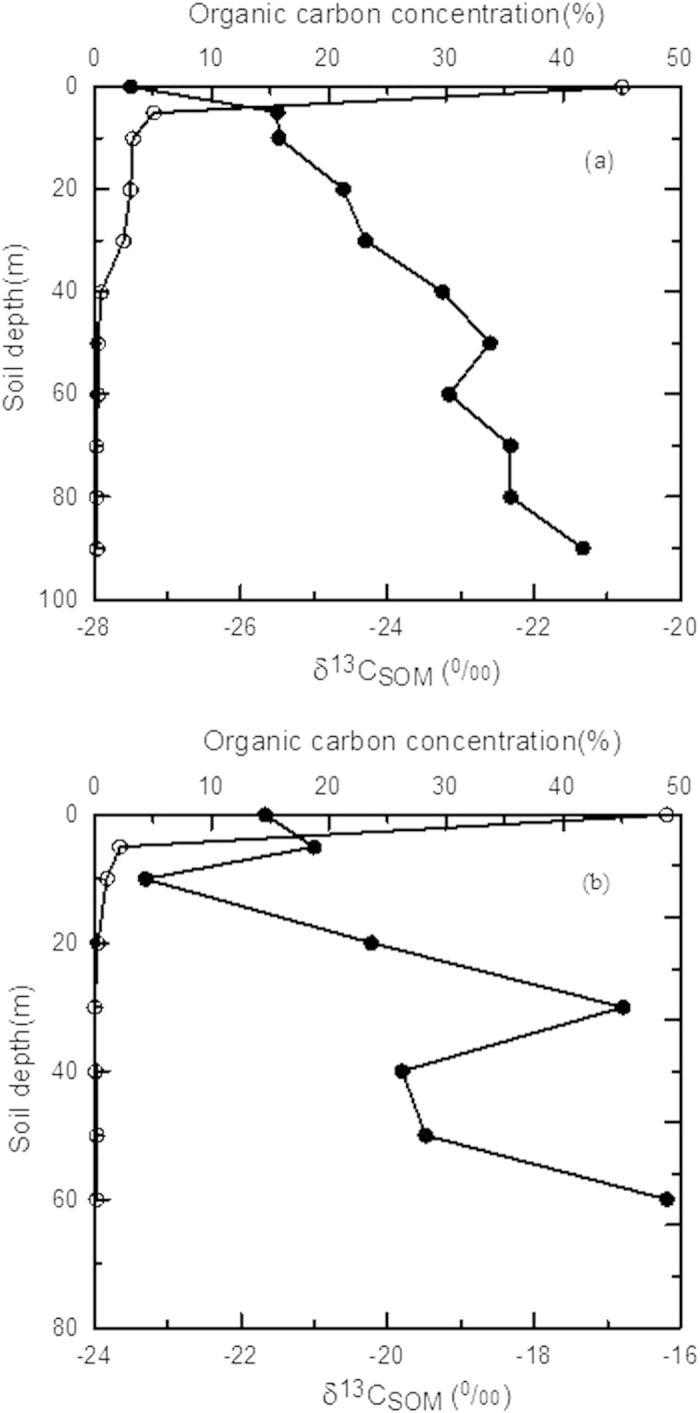
shows the vertical changes of δ^13^CSOM (closed cycles) and organic carbon concentration (open cycles). The soil profiles in Fig. 2(**a**) and (**b**) locate at 2700 m a.s.l. and 1700 m a.s.l. on the eastern slope, Mount Gongga. Note that the organic carbon concentration and δ^13^C values at depth zero in the figures are from L1 litters.

In this study, only the 60 soil profiles above 1800 m show trends of decreasing C concentration and increasing δ^13^C_SOM_ with soil depth. Figure 2a shows an example. These profiles were selected for calculation of fractionation factor α. The 15 profiles below 1800 m were omitted because the typical pattern of δ^13^C_SOM_ increase with depth was not regularly observed in these soil profiles (Fig. 2b). This indicated that the ecosystems below 1800 m experienced C_3_ to C_4_ succession, or vice versa^36^,^37^. The fractionation factor α of studied samples ranged from 1.00051 to 1.00183 with an average of 1.00118. About 80% of these samples fell between 1.0007 and 1.0017. The ε value was between −0.51 and −1.83 with an average of −1.185. Approximately 80% of sample values for ε fell between −0.75 and −1.7.

**Figure 3:**
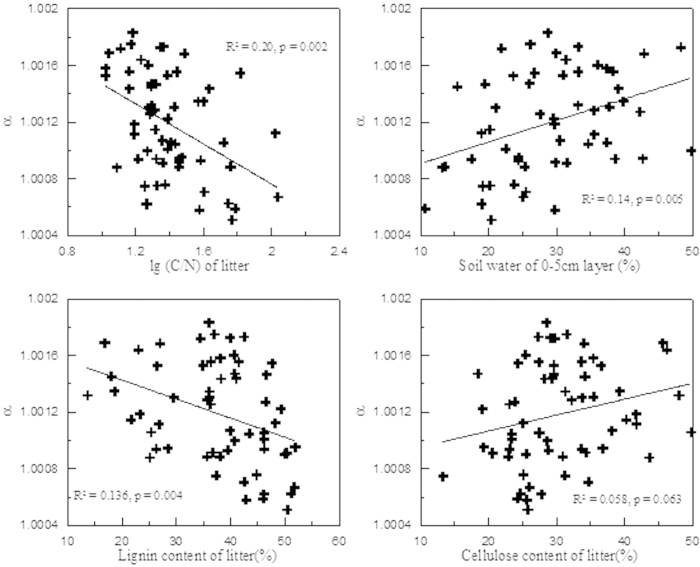
shows the relationships between α and lg(C/N), lignin content, cellulose content and soil water of 0–5 cm layer.

Initially, a series of bivariate correlation analyses were conducted to preliminarily discover the effects of different environmental and biotic factors on fractionation factor α. Figure 3 showed that water contents of 0–5 cm soil layer (W1) exerted positive impact on α (p = 0.006), while C/N and lignin content showed negative influences (p = 0.001 and = 0.004, respectively). The effects of cellulose and water contents of 5–10 cm soil layer (W2) were marginally significant (p = 0.063 and = 0.059, respectively) with positive coefficients. No significant relationship existed between α and mean annual temperature (MAT) and mean summer temperature (MST) (both p = 0.3). Afterwards, a multiple regression of α against the seven factors including C/N ratio, lignin, cellulose, W1, W2, MAT and MST was conducted using ordinary least square (OSL) estimation. This was done to find out how much variances in fractionation factor α were explained by these variables. Although the overall regression is highly significant (p = 0.000, n = 60), the 7 variables just account for 36% (R^2^) of the total variance. Considering that there are close relationships (collinearity) among variables, for examples, C/N ratio vs. lignin content (p < 0.001), W1 vs. W2 (p < 0.001), and MAT vs. MST (p < 0.001), a stepwise regression (α vs. C/N ratio, lignin, cellulose, W1, W2, MAT and MST) was run with forward direction (criteria: probability of F to enter r ≤ 0.05, probability of F to remove r ≥ 0.100), The result showed that only two variables, C/N and W1 entered the models, suggesting that they were the most significant influential factors. This regression also shows that C/N ratio of litter displayed a greater effect on α than W1 because C/N ratio entered the model earlier than W1.

OLS estimation is based on the data with characteristics of normal distribution and can describe the influence of independent variables on the mean dependent variable. For the data with characteristics of non-normal distribution, OLS no longer has the advantage of the best linear unbiased estimation (BLUE). Consequently, it is not able to effectively describe the influence of independent variables on the minimum and maximum of range of dependent variables. Instead, quantile regression overcomes the limitations of the OSL estimation and can more precisely reflect the effects of independent variables on dependent variables at different quantiles. Thus, in this study quantile regressions were run to exam the dependence of α on environmental and biotic factors in detail. It was also based on the consideration of close relationships (collinearity) among variables, in the quantile regression only three variables, C/N, W1 and MAT were taken as the predictors. The results showed the superiority of the quantile regression (Table 1). This provided more information about the influence of environmental and biotic factors on fractionation factor α compared to the multiple regression based on OSL estimation. For example, the multiple regression shows that W1 exerted a significant impact on α variance; but the quantile regression pointed out that the significant impact did not occur at quantiles 75 and 90. In addition, the multiple regression shows that fractionation factor α was independent of MAT; however, the quantile regression demonstrates that it is not always the case. For example, MAT played a significant role in fractionation factor α at quantile 10. Furthermore, although the influence of MAT on α was slight in the most cases, the influence changed with quantiles. MAT’s impact was positive at quantiles 10 50, 75 and 90, and negative at quantiles 25 (Table 1).

## Discussion

This study showed that α values tend to decrease with higher C/N ratios, higher lignin contents, and lower cellulose contents. This suggested that an increase in α is associated with higher litter quality (lower C/N ratios, lower lignin content, and higher cellulose content). Although at present no direct observation of a correlation between α and litter quality has been reported, two previous studies may indirectly support our finding. Ågren *et al*. (1996)^3^ compiled the data from Balesdent *et al*. (1993)^2^ and observed that less ^13^C enrichment occurred in soil where evergreen trees were located, which presumably had a lower litter quality. They further reported that a great ^13^C enrichment occurred in deciduous forests, which were expected to have a higher litter quality. Garten *et al*. (2000)^25^ measured δ^13^C_SOM_ in the Southern Appalachian Mountains, USA, and found that less vertical change in δ^13^C_SOM_ was associated with poorer litter quality (higher C/N ratios). The ^13^C enrichment and fractionation factor are not the same parameter, but generally, ^13^C enrichment is positively proportional to α. Thus, the two previous investigations also suggest increasing α with litter quality.

During decomposition, microbes assimilate a part of the existing organic carbon to build their bodies, and remaining organic carbon is oxidated into CO_2_ and H_2_O^12^,^13^,^38^. In the meantime, the reaction releases some energy. N is a main element of protein used for building a microbial body and a microbe also needs to absorb N while decomposing organic matter^19^,^38^. Thus, a low C/N ratio of litter helps a microbe to grow and decompose matter^23^,^24^,^39^. Lignin is generally thought to be the slowest decomposing component, although some observations contradicting this idea have been reported^22^,^40^,^41^,^42^. Low lignin contents and high cellulose contents both benefit microbial growth and decomposition of organic matter. Decomposition of organic matter is a very complex biochemical reaction. The carbon isotope fractionation factor α of the reaction is shown as follows:


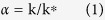


where k and k^*^ are the rate constants of the decomposition reaction involving ^12^C-substituted organic matter (or molecule) and ^13^C-substituted organic matter (or molecule), respectively. Since the decomposition rate of organic matter increases with decreasing C/N ratio and lignin content and increasing cellulose content, both the rate of decomposition involving ^13^C-substituted organic matter and the rate of decomposition involving ^12^C-substituted organic matter also increase. However, because ^13^C-substituted organic matter (or molecule) has a higher activation energy than ^12^C-substituted organic matter (or molecule), the decomposition reaction involving ^13^C-substituted organic matter increases its rate less than the reaction involving ^12^C-substituted organic matter. This feature causes α to increase with decreasing C/N ratio and lignin content and with increasing cellulose content of litter.

The second explanation for a change in α with litter quality is that the properties and compositions of microbial decomposer communities are associated with litter quality^43^,^44^,^45^. Different microbes have different metabolic pathways even when they decompose the same organic compound^43^,^45^,^46^, and the extent of isotope fractionation during decomposition may be tightly related to the metabolic pathways of microbes^43^. For example, Morasch *et al*. (2001) observed a greater hydrogen isotope fractionation for toluene degradation in growth experiments with the aerobic bacterium *P. putida* mt-2 and a less fractionation in toluene degradation by the anaerobic bacteria^47^.

High soil water content could lead to the formation of an anaerobic environment that limits microbial growth and decomposition of organic matter. However, this study shows that higher soil water content is related to a greater carbon isotope fractionation. The effect of water availability on isotope fractionation is also associated with compositions of microbial communities. Microbial communities in aerobic environments differ from those in anaerobic environments. As mentioned above, different microbes could use different metabolic pathways to decompose the same organic compound, thus, aerobic microbes could produce a different isotope fractionation from that produced by anaerobic microbes. For example, Griebler *et al*. (2004) did not observe a significant carbon isotope fractionation during mineralization of 1,2,4-trichlorobenzene by the aerobic strain *Pseudomonas* sp. P51, which used a dioxygenase for the initial enzymatic reaction^48^. In contrast, carbon isotope enrichment factors were between −3.1‰ and −3.7‰ for the degradation of 1,2,3- and 1,2,4-trichlorobenzene by the anaerobic strain *Dehalococcoide* sp.

For a pure chemical reaction, the magnitude of the kinetic fractionation factor α is dependent on temperature and difference of activation energy (ΔQ) between heavy and light isotopically substituted molecules of the reactant^49^. Parameter α decreases with an increase in temperature when ΔQ remains constant^47^,^49^. However, for isotope fractionation in a biochemical reaction, in addition to being affected by temperature and the ΔQ, it also depends on the activity of enzymes and microbes because an increase in activity of enzymes and microbes benefits enhanced decay rate, causing a greater isotopic fractionation during decomposition^50^,^51^. Rising temperature generally leads to elevated enzyme and microbe activities^44^,^45^,^46^, leading to increase in isotope fractionation. Coleman *et al*. (1981) found that undefined cultures of methane-oxidizing bacteria displayed greater carbon isotope fractionation at 30 °C than 11.5 °C^52^. However, current study found that temperature had no effect on α in most cases. The potential mechanism is that with decreasing temperature, the α induced by a pure chemical process would increase whereas the α induced by enzyme and microbe would decrease owing to a decrease in enzyme and microbe activities. Most probably the α induced by enzyme and microbe offset the α induced by a pure chemical process. This eventually rendered temperature to exhibit no impact on isotope fractionation.

The quantile regression showed that fractionation factor α at high quantiles was independent of soil water and MAT, suggesting that a great isotope fractionation during SOM decomposition was associated with high litter quality which positively influenced α variance during decomposition (Table 1). Organic matter with high quality often maintains high decay rate, consequently, a great α is produced. Table 1 shows that α variance at low quantiles was affected by both litter quality and environmental factors, indicating that the decomposition of organic matter with low quality depended on environmental conditions, especially soil water status. Quantile regression further demonstrates that the impact of temperature on fractionation was positive in most cases although it was not slight. The finding suggests that the α induced by enzyme and microbe was slightly bigger than the α induced by a pure chemical process.

Although the multiple regression of α against 7 variables including C/N, lignin, cellulose, W1, W2, MAT and MST is highly significant, only 36% of the α variance can be explained by these environmental and biotic factors. Three potential reasons were responsible for such a low amount of explanation. 1) In this study, we calculated fractionation factor α of each soil profile based on the approach of Poage and Feng (2004)^13^. However, they explained the ^13^C profile solely by fractionation of organic matter during decomposition and ignored the other hypotheses such as different isotopic signature of root litter compared to surface litter or temporal changes of isotope composition of vegetal inputs progressively incorporated into the soil^13^. 2) Soil layer is too thin, even less than 20 cm, at some sampling sites, thus, the obtained α values in these profiles with thin soil layer may be not very reliable due to limited δ^13^C_SOM_ data. 3) The soil water contents used in this study were measured in the dry season, while fractionation during organic matter decomposition is dependent of long-term soil water conditions. Thus, further studies are needed in the regard.

Although there were some sorts of limitations mentioned above, we are fairly confident that the α values obtained in current study reflect the actual values of the isotope fractionation factor during organic matter decomposition in the ecosystem studied. This confidence is based on the following two facts: 1) the average δ^13^C values did not display differences among the bulk individual, leaf, stem and root in our other study conducted recently on 22 C_3_ plants and 6 C_4_ plants (unpublished). 2) A previous investigation conducted in the same study area showed that the mean δ^13^C difference between 0–5 cm soil layer and vegetation was very big, 2.87‰, while the mean δ^13^C differences between 0–5 cm and 5–10 cm and 10–20 cm soil layer were very small, 0.17‰ and 0.62‰, respectively^36^. The 2.87‰ difference between 0–5 cm layer and vegetation showed the combined contributions of carbon isotope fractionation during organic matter decomposition and the temporal changes of plants δ^13^C induced mainly by the δ^13^C decrease in atmospheric CO_2_ since the industrial revolution. In present study, Δδ^13^C_SOM_ in Eqn. 4 is the δ^13^C difference between mineral soil samples and the surface mineral soil (0–5 cm depth), thus, the effect of δ^13^C temporal changes of plant inputs on the α calculation should be small and could be neglected. On the other hand, all α values obtained were very small with an average of 1.00118. If the temporal changes of plants δ^13^C contained in the α calculation, the α values obtained will be probably negative, which would be inconsistent with the actual situation.

In conclusion, this study shows that the magnitude of isotope fractionation during SOM decomposition was related to biotic and environmental factors. Litter quality and soil water content both had positive impact on α whereas temperature displayed no effect.

## Methods

### Study site

Mount Gongga is located in the southeastern side of the Tibetan Plateau (101°30΄ ~ 102°10΄E, 29°20΄ ~ 30°00΄N). There are remarkable differences in terrain and climate between the eastern and western slopes of this area. We selected the eastern slope of Mount Gongga as a study site because it consists of many climate types, diverse ecological systems, and stable vegetation types ranging from tropical, subtropical to cold zone, and relatively little human disturbance. The eastern slope belongs to an alpine gorge landform. The altitude of the eastern slope of Mount Gongga varies from 1100 m a.s.l. (Dadu River valley) to 7600 m a.s.l., and its climate is warm and dry at low elevations and cold and moist at high elevations. On the slope, temperature decreases and precipitation may increase with increasing altitude; this feature is based on the records of two meteorological observatories on the slope^53^.

An intact and continuous vertical vegetation spectrum can be observed along the eastern slope of Mount Gongga. It consists of subtropical evergreen broad-leaved vegetation (1100–2200 m, including a semi-arid valley with shrubs and grasses (<1500 m), evergreen broad-leaved forests, and deciduous broad-leaved forests), temperate coniferous and broad-leaved mixed forests (2200–2800 m), frigid dark coniferous forests (2800–3600 m), alpine subfrigid shrub and meadow vegetation (3600–4200 m), alpine frigid meadow vegetation (4200–4600 m), alpine frigid sparse grasses and a desert zone (4600–4800 m), and a high-altitude alpine ice-and-snow zone (>4900 m). The vertical distribution of soil on the eastern slope of Mount Gongga is also very pronounced, and a continuous soil sequence occurs from 1100 m to 4900 m. It consists of yellow-red soil (luvisols) (<1500 m), yellow-brown soil (luvisols) (1500–1800 m), brown soil (1800–2200 m) (luvisols), dark-brown soil (luvisols) (2200–2800 m), dark-brown forest soil (luvisols) (2800–3600 m), black mattic soil (cambisols) (3600–4200 m), mattic soil (luvisols) (4200–4600 m), and chilly desert soil (cryosols) (>4600 m)^54^.

### Sample collection

A vertical transect spanning from 1200 m a.s.l. to 4500 m a.s.l., across five vegetation types and seven soil types, was set on the eastern slope of Mount Gongga. In August 2004, samples (including plant leaves, litter, and soil) were collected along the transect at intervals of about 100 m. The method of plant sampling was described in previous papers^54^,^55^. At the most sampling sites, we set three plots (0.5 m × 0.5 m) within a 200 m^2^ area. All aboveground litters within a plot were collected, and then, a soil profile was dug to the weathered rock. The depth of a soil profile depended on the depth of the weathered rock, and most profiles had the depth of 40 cm to 50 cm. In total, 27 sites with 75 plots and 75 soil profiles were sampled along the transect. Organic layers above mineral soil were defined as “litter.” Depth zero refers to the top of the mineral horizon. Litter samples (0.25 m^2^) were separated into one to four layers, depending on the humus type. Layers were separated and defined by visual aspect according to Kubiena (1953)^56^. The first layer (L1) contains entire leaves remaining from the last fall. The second layer (L2) consists of partial leaves and partially decomposed small wood. The third layer (F) consists of small pieces (<10 mm) of decomposed leaves and small wood. The fourth layer is the dark, fine, moders and mors. Mineral soil was collected at 5 cm intervals down to a 10 cm depth, after which it was collected at 10 cm intervals down to the bottom of the soil profile.

### Measurements of soil water content

Soil water content for each of three layers (0–5 cm, 5–10 cm, and 10–20 cm) was determined by comparing the weight of wet and dry soils. Wet soil, the intact natural soil, was oven-dried at 105 °C until the weight did not change anymore.

### Measurements of δ^13^C and C concentration of soil organic matter

Soil was oven-dried at 50 °C for 24 h; afterward, stones and plant residues in soil were removed; finally, soil was ground and filtered through a 2 mm sieve. About 3 g of soil consisting of particles less than 2 mm in diameter was immersed by excessive HCl (1 mol/l) for 24 h to remove carbonate, and then washed to neutrality by distilled water^57^. Finally, the soil was oven-dried at 50 °C and ground into a fine powder. Measurements of δ^13^C and C concentration of SOM were determined on a Delta^Plus^XP mass spectrometer (Thermo Scientific, Bremen, Germany) that was coupled with an elemental analyzer in continuous flow mode. The elemental analyzer (FlashEA 1112; CE Instruments,Wigan, UK) combustion temperature was 1020 °C.

The carbon isotopic ratios are reported in standard notation, relative to the V-PDB standard. The standard deviations for measurements of soil *δ*^13^C and soil C concentrations were less than 0.2‰ and 0.1%, respectively.

### Measurements of C and N concentrations in litter

The litter samples (L1) were oven-dried at 65 °C and ground into a fine powder. The measurements of C and N concentrations were conducted on an elemental analyzer (FlashEA 1112; CE Instruments, Wigan, UK). The standard deviations for measurements of litter C and N both were less than 0.1%.

### Measurements of fiber and lignin in litter

Fiber is the insoluble residue in litter after removing fat, starch, protein, and sugar by acid detergent. It includes cellulose and lignin. Lignin is the insoluble residue after dissolving fiber by sulfuric acid.

Fiber was obtained through the following steps. The first step was to pour 100 ml of hot acid detergent in a beaker with 1 g of litter (previously ground and sieved through a 2 mm sieve), covered the condensing ball, opened the cooling water, quick heated the beaker to a boiling state, and then maintained boiling for 60 min. The second step was to pour the solution into a filter crucible and then vacuumed and filtered the solution so that all acid was removed. The third step was to wash the residues left in the filter crucible two times with 40 ml acetone and then filter the solution until the filtrate was transparent. Each wash lasted 3–5 min. The final step was to place the filter crucible with residues into a ventilate cabinet until all of the acetone evaporated, dried the filter crucible with residues for 4 h at 105 °C, and then weighed the filter crucible with residues and recorded the mass. We denoted the recorded mass as m_2_. The fiber content (%) was calculated by the following equation (1):


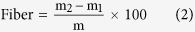


where m is the sample mass (1 g in this measurement), m_1_ is the crucible mass, and m_2_ is the total mass of the crucible and residue.

Lignin was obtained through the following steps. The first step was to place the above residue into a 50 ml beaker, then 12.0 mol L^−1^ sulfuric acid was poured into the beaker, and let the acid digest for 3 h at 20–25 °C. The second step was to pour the solution into a filter crucible, vacuum and filter the solution so that all of the acid was removed, and then repeatedly washed the residual material with hot water until its pH equaled 7. The residue was lignin, and the amount of cellulose was the difference between the fiber and lignin amounts.

### Definitions and basic equations

The isotope fractionation factor α indicates the degree of isotope fractionation; a larger *α* value means a greater fractionation. In Poage and Feng (2004)^13^, the carbon isotope fractionation factor *α* during decomposition of organic carbon was defined as


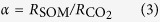


where *R*_SOM_ and *R*_CO2_ are the ^13^C/^12^C ratios of organic carbon (substrate) and respired CO_2_ (product), respectively.

In this study, we calculated the carbon isotope fractionation factor α of decomposing organic matter by using the data of δ^13^C_SOM_ of soil profiles. It must be noted that only the soil profiles from sites with constant C_3_ or C_4_ vegetation can be used to do the calculation. The first step to obtaining *α* value of each soil profile was to calculate the δ^13^C difference (Δδ^13^C_SOM_) between mineral soil samples and the surface mineral soil (0–5 cm depth). The second step was to calculate ln(*ρ*/*ρ*_0_), where *ρ*_0_ is the C density of the surface mineral soil, and *ρ* denotes the C density of mineral soil samples^13^. We used C concentration instead of C density in this calculation, and generally, the alternative calculation does not cause big errors (personal communication with Feng). The third step was to plot Δδ^13^C_SOM_ and ln(*ρ*/*ρ*_0_). Finally, *α* is calculated from the slope of the following linear equation^13^:


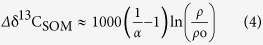


**Figure 4:**
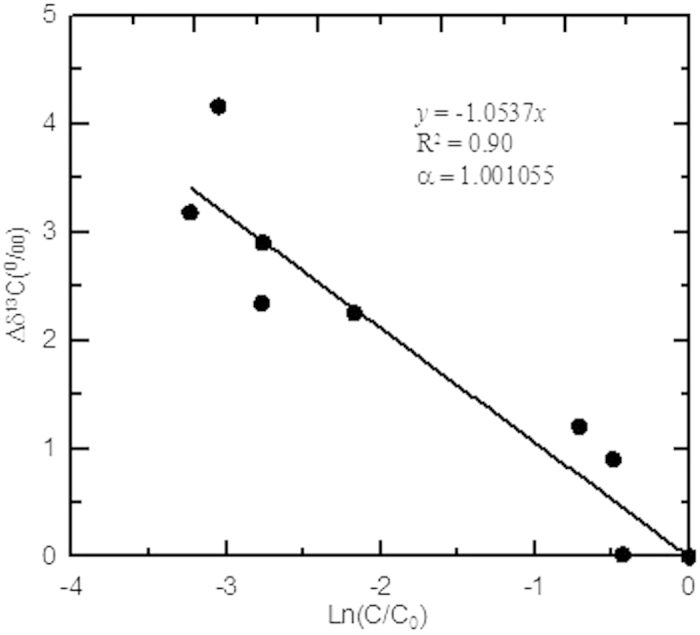
shows how to obtain α value of a soil profile located at 2700 m a.s.l. on the eastern slope, Mount Gongga. The α value was estimated indirectly as the slope of the linear equation between Δδ^13^C and ln(C/C_0_).

Figure 4 shows an example of obtaining α for a soil profile located at 2700 m a.s.l. on the eastern slope of Mount Gongga.

Ecologists often use isotope discrimination, ε, to describe fractionation of a biochemical process:


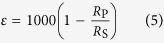


where *R*_p_ is the ^13^C/^12^C ratio of the product (CO_2_) and *R*_S_ the source (substrate) ratio. Comparing Eqns. 3 and 5, one can obtain Eqn. 6:


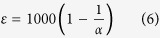


Eqn. 6 shows that a larger α value means a greater absolute ε value, which indicates a greater isotope fractionation.

### Statistical analyses

In this study, a series of bivariate correlation analyses were first conducted to preliminarily exam the effects of different environmental and biotic factors (including C/N ratio, lignin, cellulose, W1, W2, MAT and MST) on fractionation factor α, then a stepwise regression of α vs. the 7 variables was run with forward direction (criteria: probability of F to enter r ≤ 0.05, probability of F to remove r ≥ 0.100) so that the most important influential factors could be determined. In order to effectively describe the influence of environmental and biotic variables on the minimum and maximum of range of fractionation factors, quantile regression of α vs. C/N, W1 and MAT was carried out. We used the statistical software SPSS 11.0 (SPSS Inc., Chicago, IL, USA) for the analyses of correlation and stepwise regression, and the Stata/SE120 for Windows (StataCorp LP, USA) for the quantile regression.

## Additional Information

**How to cite this article**: Wang, G. *et al*. Effects of environmental and biotic factors on carbon isotopic fractionation during decomposition of soil organic matter. *Sci. Rep*. **5**, 11043; doi: 10.1038/srep11043 (2015).

## Figures and Tables

**Table 1:** shows the results of multiple regressions based on OSL and quantile regressions of α against the C/N, W1 and MAT.

Variables	OLS	Quantile regression				
		Quant10	Quant25	Quant50	Quant75	Quant90
Constant	1.00156^***^ (3063.52)	1.00081^***^ (3048.89)	1.00128^***^ (2213.83)	1.00144^***^ (2100.46)	1.00234^***^ (1963.74)	1.00215^***^ (2874.42)
C/N ratio	−0.000693^***^ (−3.53)	−0.000462^**^ (−2.35)	−0.00060^**^ (−2.23)	−0.000694^**^ (−2.43)	−0.001011^**^ (−3.30)	−0.000671^**^ (−3.21)
W1	0.000020^**^ (2.58)	0.0000118^**^ (2.35)	0.000013^*^ (1.92)	0.000017^**^ (2.33)	0.000010 (1.34)	5.47e-06 (1.03)
MAT	−2.68e-06 (−0.03)	0.0000181^*^ (1.99)	−0.000016 (−1.28)	1.32e-06 (0.10)	5.27e-06 (0.37)	9.73e-06 (1.0)
R^2^	0.27	0.25	0.19	0.22	0.15	0.15

Notes: ^*^, ^**^ and ^***^ indicate significant effects at p < 0.1, 0.05 and 0.01 levels, respectively. The numbers shown in the table are the coefficients and t values (in brackets), respectively.
